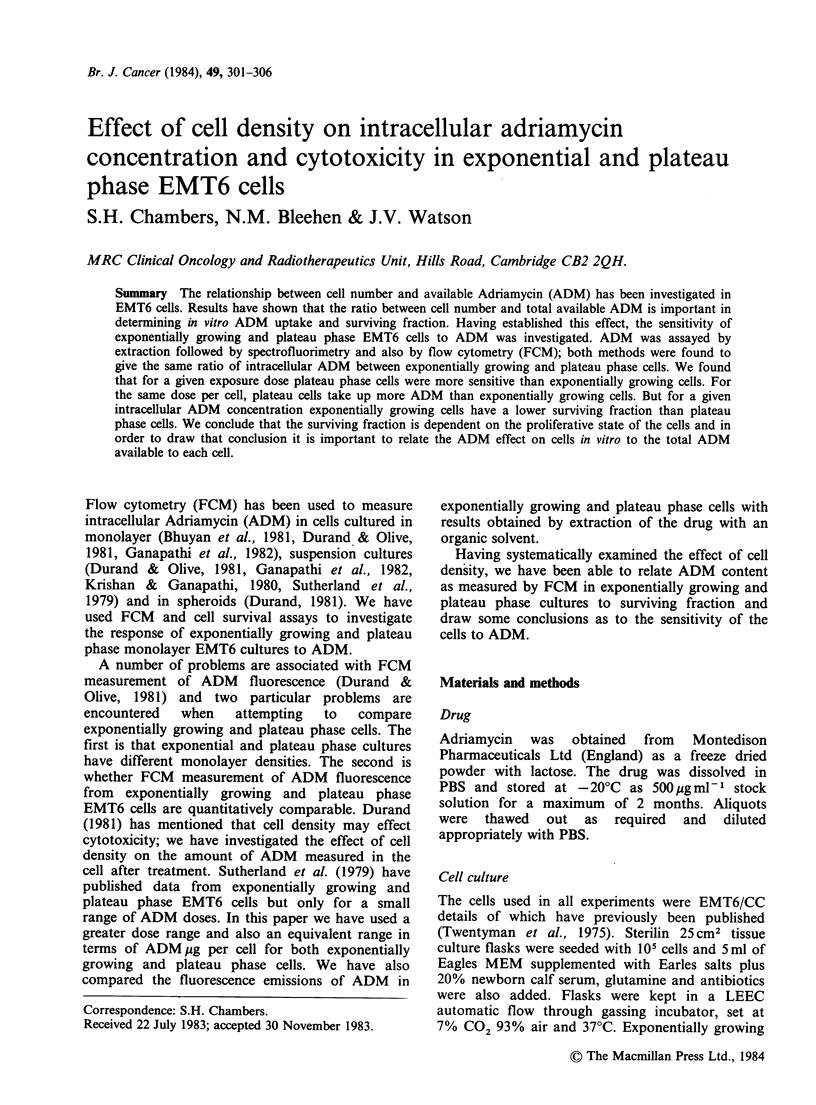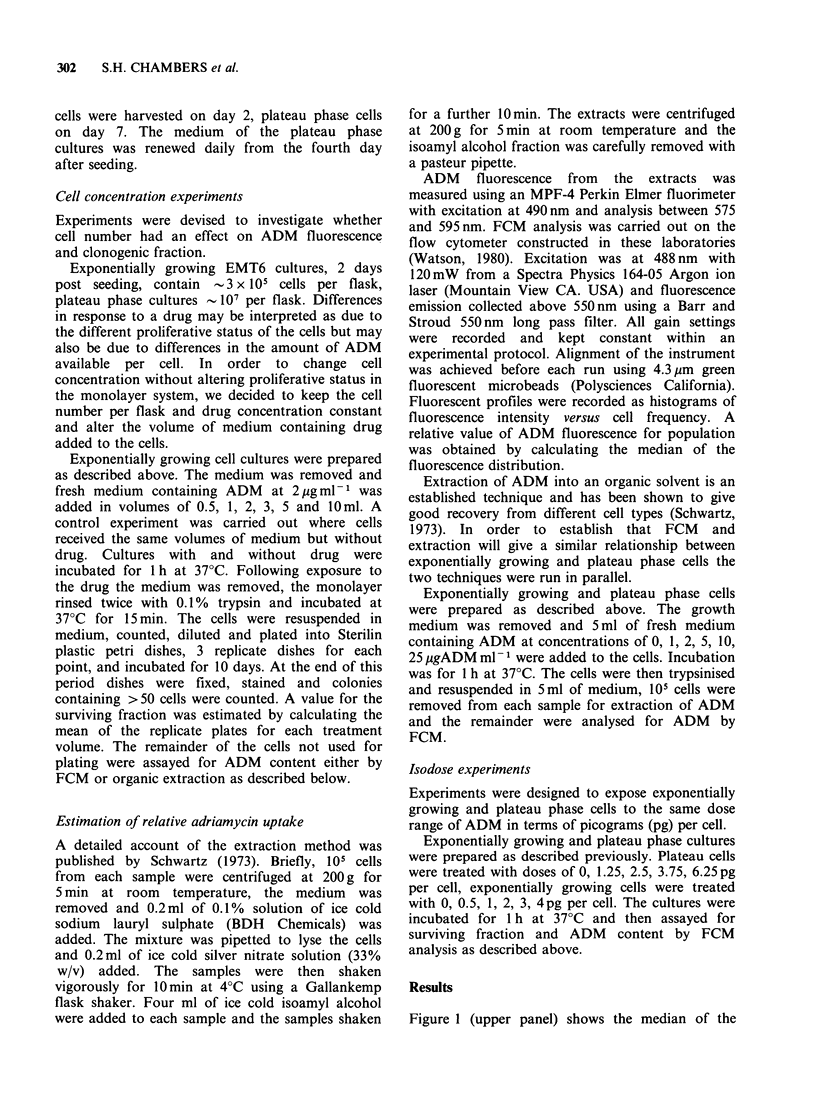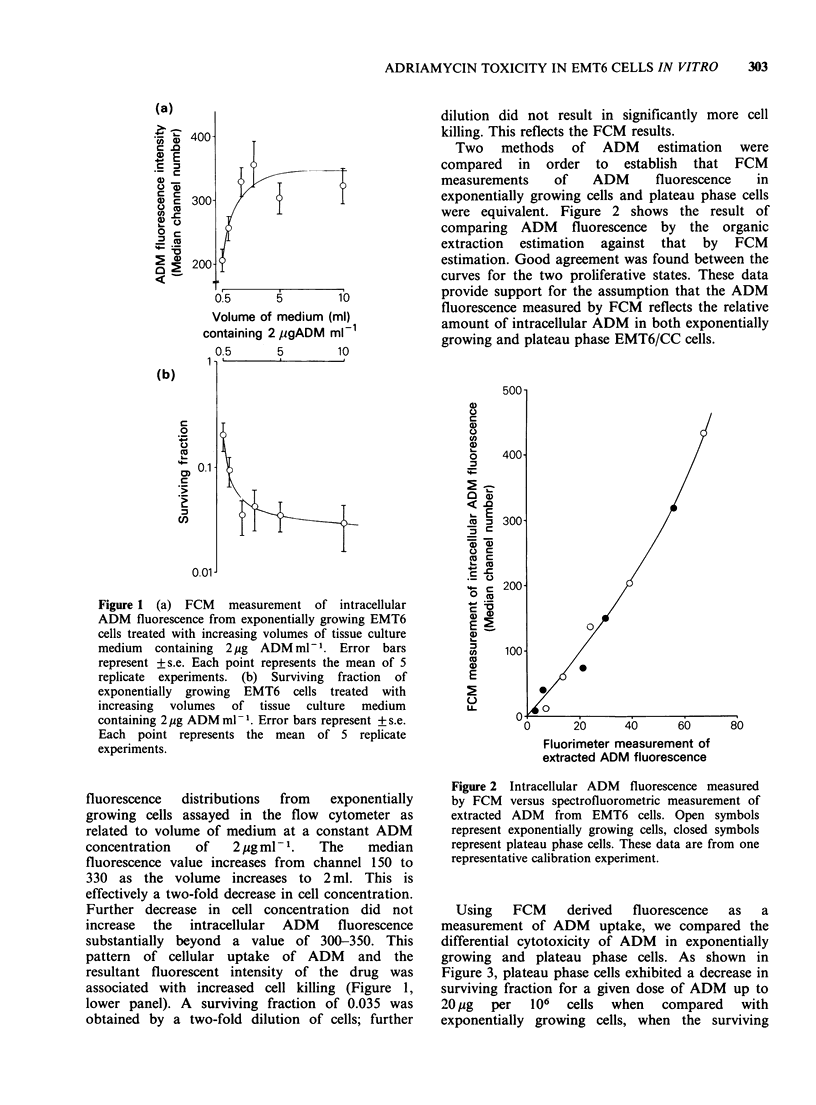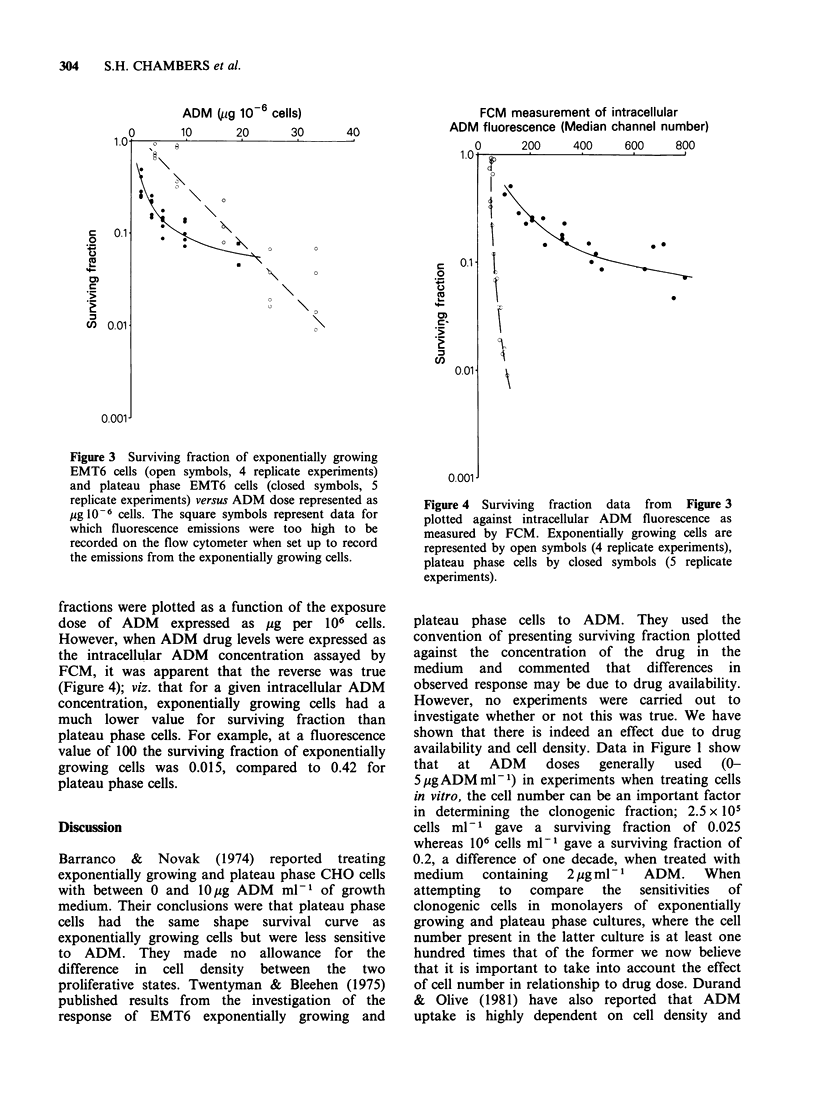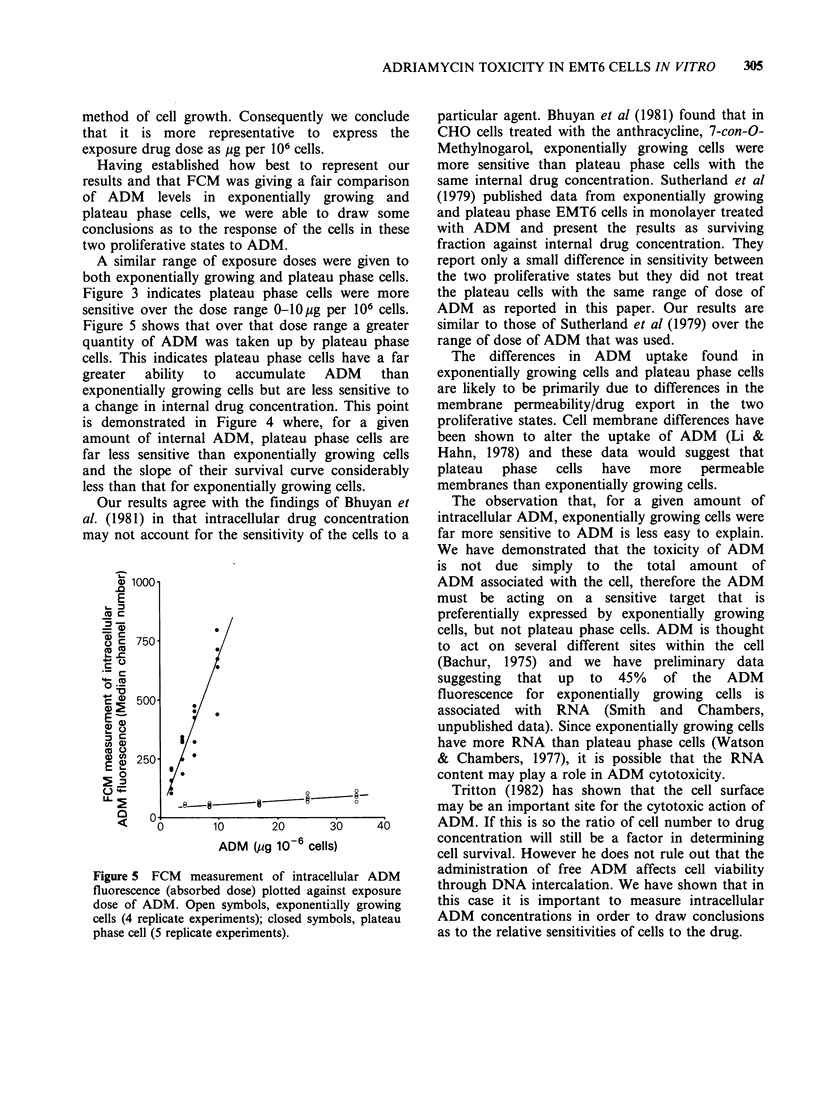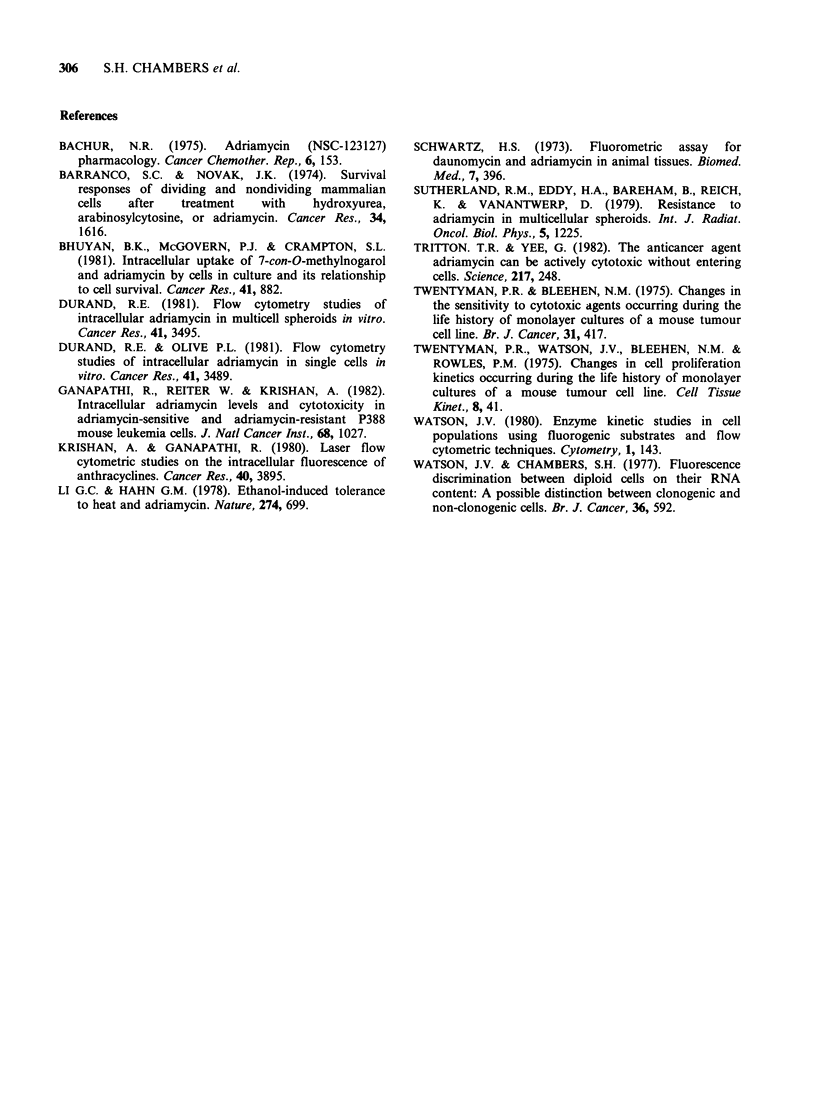# Effect of cell density on intracellular adriamycin concentration and cytotoxicity in exponential and plateau phase EMT6 cells.

**DOI:** 10.1038/bjc.1984.47

**Published:** 1984-03

**Authors:** S. H. Chambers, N. M. Bleehen, J. V. Watson

## Abstract

The relationship between cell number and available Adriamycin (ADM) has been investigated in EMT6 cells. Results have shown that the ratio between cell number and total available ADM is important in determining in vitro ADM uptake and surviving fraction. Having established this effect, the sensitivity of exponentially growing and plateau phase EMT6 cells to ADM was investigated. ADM was assayed by extraction followed by spectrofluorimetry and also by flow cytometry (FCM); both methods were found to give the same ratio of intracellular ADM between exponentially growing and plateau phase cells. We found that for a given exposure dose plateau phase cells were more sensitive than exponentially growing cells. For the same dose per cell, plateau cells take up more ADM than exponentially growing cells. But for a given intracellular ADM concentration exponentially growing cells have a lower surviving fraction than plateau phase cells. We conclude that the surviving fraction is dependent on the proliferative state of the cells and in order to draw that conclusion it is important to relate the ADM effect on cells in vitro to the total ADM available to each cell.


*Br. J. Cancer* (1984), **49**, 301–306

### Effect of cell density on intracellular adriamycin concentration and cytotoxicity in exponential and plateau phase EMT6 cells

S.H. Chambers, N.M. Bleehen & J.V. Watson

*MRC Clinical Oncology and Radiotherapeutics Unit, Hills Road, Cambridge CB2 2QH.*

**Summary** The relationship between cell number and available Adriamycin (ADM) has been investigated in EMT6 cells. Results have shown that the ratio between cell number and total available ADM is important in determining *in vitro* ADM uptake and surviving fraction. Having established this effect, the sensitivity of exponentially growing and plateau phase EMT6 cells to ADM was investigated. ADM was assayed by extraction followed by spectrofluorimetry and also by flow cytometry (FCM); both methods were found to give the same ratio of intracellular ADM between exponentially growing and plateau phase cells. We found that for a given exposure dose plateau phase cells were more sensitive than exponentially growing cells. For the same dose per cell, plateau cells take up more ADM than exponentially growing cells. But for a given intracellular ADM concentration exponentially growing cells have a lower surviving fraction than plateau phase cells. We conclude that the surviving fraction is dependent on the proliferative state of the cells and in order to draw that conclusion it is important to relate the ADM effect on cells *in vitro* to the total ADM available to each cell.

Flow cytometry (FCM) has been used to measure intracellular Adriamycin (ADM) in cells cultured in monolayer (Bhuyan *et al.*, 1981, Durand & Olive, 1981, Ganapathi *et al.*, 1982), suspension cultures (Durand & Olive, 1981, Ganapathi *et al.*, 1982, Krishan & Ganapathi, 1980, Sutherland *et al.*, 1979) and in spheroids (Durand, 1981). We have used FCM and cell survival assays to investigate the response of exponentially growing and plateau phase monolayer EMT6 cultures to ADM.

A number of problems are associated with FCM measurement of ADM fluorescence (Durand & Olive, 1981) and two particular problems are encountered when attempting to compare exponentially growing and plateau phase cells. The first is that exponential and plateau phase cultures have different monolayer densities. The second is whether FCM measurement of ADM fluorescence from exponentially growing and plateau phase EMT6 cells are quantitatively comparable. Durand (1981) has mentioned that cell density may effect cytotoxicity; we have investigated the effect of cell density on the amount of ADM measured in the cell after treatment. Sutherland *et al.* (1979) have published data from exponentially growing and plateau phase EMT6 cells but only for a small range of ADM doses. In this paper we have used a greater dose range and also an equivalent range in terms of ADM $\mu$g per cell for both exponentially growing and plateau phase cells. We have also compared the fluorescence emissions of ADM in exponentially growing and plateau phase cells with results obtained by extraction of the drug with an organic solvent.

Having systematically examined the effect of cell density, we have been able to relate ADM content as measured by FCM in exponentially growing and plateau phase cultures to surviving fraction and draw some conclusions as to the sensitivity of the cells to ADM.

Correspondence: S.H. Chambers.
Received 22 July 1983; accepted 30 November 1983.

### Materials and methods

### *Drug*

Adriamycin was obtained from Montedison Pharmaceuticals Ltd (England) as a freeze dried powder with lactose. The drug was dissolved in PBS and stored at $-20°C$ as $500\,\mu g\,ml^{-1}$ stock solution for a maximum of 2 months. Aliquots were thawed out as required and diluted appropriately with PBS.

### *Cell culture*

The cells used in all experiments were EMT6/CC details of which have previously been published (Twentyman *et al.*, 1975). Sterilin $25\,cm^2$ tissue culture flasks were seeded with $10^5$ cells and 5 ml of Eagles MEM supplemented with Earles salts plus 20% newborn calf serum, glutamine and antibiotics were also added. Flasks were kept in a LEEC automatic flow through gassing incubator, set at 7% $CO_2$ 93% air and 37°C. Exponentially growing

© The Macmillan Press Ltd., 1984

cells were harvested on day 2, plateau phase cells on day 7. The medium of the plateau phase cultures was renewed daily from the fourth day after seeding.

*Cell concentration experiments*

Experiments were devised to investigate whether cell number had an effect on ADM fluorescence and clonogenic fraction.

Exponentially growing EMT6 cultures, 2 days post seeding, contain $\sim 3 \times 10^5$ cells per flask, plateau phase cultures $\sim 10^7$ per flask. Differences in response to a drug may be interpreted as due to the different proliferative status of the cells but may also be due to differences in the amount of ADM available per cell. In order to change cell concentration without altering proliferative status in the monolayer system, we decided to keep the cell number per flask and drug concentration constant and alter the volume of medium containing drug added to the cells.

Exponentially growing cell cultures were prepared as described above. The medium was removed and fresh medium containing ADM at $2\,\mu g\,ml^{-1}$ was added in volumes of 0.5, 1, 2, 3, 5 and 10 ml. A control experiment was carried out where cells received the same volumes of medium but without drug. Cultures with and without drug were incubated for 1 h at 37°C. Following exposure to the drug the medium was removed, the monolayer rinsed twice with 0.1% trypsin and incubated at 37°C for 15 min. The cells were resuspended in medium, counted, diluted and plated into Sterilin plastic petri dishes, 3 replicate dishes for each point, and incubated for 10 days. At the end of this period dishes were fixed, stained and colonies containing $>50$ cells were counted. A value for the surviving fraction was estimated by calculating the mean of the replicate plates for each treatment volume. The remainder of the cells not used for plating were assayed for ADM content either by FCM or organic extraction as described below.

*Estimation of relative adriamycin uptake*

A detailed account of the extraction method was published by Schwartz (1973). Briefly, $10^5$ cells from each sample were centrifuged at 200 g for 5 min at room temperature, the medium was removed and 0.2 ml of 0.1% solution of ice cold sodium lauryl sulphate (BDH Chemicals) was added. The mixture was pipetted to lyse the cells and 0.2 ml of ice cold silver nitrate solution (33% w/v) added. The samples were then shaken vigorously for 10 min at 4°C using a Gallankemp flask shaker. Four ml of ice cold isoamyl alcohol were added to each sample and the samples shaken for a further 10 min. The extracts were centrifuged at 200 g for 5 min at room temperature and the isoamyl alcohol fraction was carefully removed with a pasteur pipette.

ADM fluorescence from the extracts was measured using an MPF-4 Perkin Elmer fluorimeter with excitation at 490 nm and analysis between 575 and 595 nm. FCM analysis was carried out on the flow cytometer constructed in these laboratories (Watson, 1980). Excitation was at 488 nm with 120 mW from a Spectra Physics 164-05 Argon ion laser (Mountain View CA. USA) and fluorescence emission collected above 550 nm using a Barr and Stroud 550 nm long pass filter. All gain settings were recorded and kept constant within an experimental protocol. Alignment of the instrument was achieved before each run using 4.3 $\mu$m green fluorescent microbeads (Polysciences California). Fluorescent profiles were recorded as histograms of fluorescence intensity *versus* cell frequency. A relative value of ADM fluorescence for population was obtained by calculating the median of the fluorescence distribution.

Extraction of ADM into an organic solvent is an established technique and has been shown to give good recovery from different cell types (Schwartz, 1973). In order to establish that FCM and extraction will give a similar relationship between exponentially growing and plateau phase cells the two techniques were run in parallel.

Exponentially growing and plateau phase cells were prepared as described above. The growth medium was removed and 5 ml of fresh medium containing ADM at concentrations of 0, 1, 2, 5, 10, $25\,\mu g$ADM $ml^{-1}$ were added to the cells. Incubation was for 1 h at 37°C. The cells were then trypsinised and resuspended in 5 ml of medium, $10^5$ cells were removed from each sample for extraction of ADM and the remainder were analysed for ADM by FCM.

*Isodose experiments*

Experiments were designed to expose exponentially growing and plateau phase cells to the same dose range of ADM in terms of picograms (pg) per cell.

Exponentially growing and plateau phase cultures were prepared as described previously. Plateau cells were treated with doses of 0, 1.25, 2.5, 3.75, 6.25 pg per cell, exponentially growing cells were treated with 0, 0.5, 1, 2, 3, 4 pg per cell. The cultures were incubated for 1 h at 37°C and then assayed for surviving fraction and ADM content by FCM analysis as described above.

### Results

Figure 1 (upper panel) shows the median of the

**Figure 1** (a) FCM measurement of intracellular ADM fluorescence from exponentially growing EMT6 cells treated with increasing volumes of tissue culture medium containing $2\,\mu g$ ADM $ml^{-1}$. Error bars represent ±s.e. Each point represents the mean of 5 replicate experiments. (b) Surviving fraction of exponentially growing EMT6 cells treated with increasing volumes of tissue culture medium containing $2\,\mu g$ ADM $ml^{-1}$. Error bars represent ±s.e. Each point represents the mean of 5 replicate experiments.

fluorescence distributions from exponentially growing cells assayed in the flow cytometer as related to volume of medium at a constant ADM concentration of $2\,\mu g\,ml^{-1}$. The median fluorescence value increases from channel 150 to 330 as the volume increases to 2 ml. This is effectively a two-fold decrease in cell concentration. Further decrease in cell concentration did not increase the intracellular ADM fluorescence substantially beyond a value of 300–350. This pattern of cellular uptake of ADM and the resultant fluorescent intensity of the drug was associated with increased cell killing (Figure 1, lower panel). A surviving fraction of 0.035 was obtained by a two-fold dilution of cells; further dilution did not result in significantly more cell killing. This reflects the FCM results.

Two methods of ADM estimation were compared in order to establish that FCM measurements of ADM fluorescence in exponentially growing cells and plateau phase cells were equivalent. Figure 2 shows the result of comparing ADM fluorescence by the organic extraction estimation against that by FCM estimation. Good agreement was found between the curves for the two proliferative states. These data provide support for the assumption that the ADM fluorescence measured by FCM reflects the relative amount of intracellular ADM in both exponentially growing and plateau phase EMT6/CC cells.

**Figure 2** Intracellular ADM fluorescence measured by FCM versus spectrofluorometric measurement of extracted ADM from EMT6 cells. Open symbols represent exponentially growing cells, closed symbols represent plateau phase cells. These data are from one representative calibration experiment.

Using FCM derived fluorescence as a measurement of ADM uptake, we compared the differential cytotoxicity of ADM in exponentially growing and plateau phase cells. As shown in Figure 3, plateau phase cells exhibited a decrease in surviving fraction for a given dose of ADM up to $20\,\mu g$ per $10^6$ cells when compared with exponentially growing cells, when the surviving

**Figure 3** Surviving fraction of exponentially growing EMT6 cells (open symbols, 4 replicate experiments) and plateau phase EMT6 cells (closed symbols, 5 replicate experiments) *versus* ADM dose represented as $\mu g\,10^{-6}$ cells. The square symbols represent data for which fluorescence emissions were too high to be recorded on the flow cytometer when set up to record the emissions from the exponentially growing cells.

**Figure 4** Surviving fraction data from **Figure 3** plotted against intracellular ADM fluorescence as measured by FCM. Exponentially growing cells are represented by open symbols (4 replicate experiments), plateau phase cells by closed symbols (5 replicate experiments).

fractions were plotted as a function of the exposure dose of ADM expressed as μg per $10^6$ cells. However, when ADM drug levels were expressed as the intracellular ADM concentration assayed by FCM, it was apparent that the reverse was true (Figure 4); *viz.* that for a given intracellular ADM concentration, exponentially growing cells had a much lower value for surviving fraction than plateau phase cells. For example, at a fluorescence value of 100 the surviving fraction of exponentially growing cells was 0.015, compared to 0.42 for plateau phase cells.

### Discussion

Barranco & Novak (1974) reported treating exponentially growing and plateau phase CHO cells with between 0 and 10 μg ADM $ml^{-1}$ of growth medium. Their conclusions were that plateau phase cells had the same shape survival curve as exponentially growing cells but were less sensitive to ADM. They made no allowance for the difference in cell density between the two proliferative states. Twentyman & Bleehen (1975) published results from the investigation of the response of EMT6 exponentially growing and plateau phase cells to ADM. They used the convention of presenting surviving fraction plotted against the concentration of the drug in the medium and commented that differences in observed response may be due to drug availability. However, no experiments were carried out to investigate whether or not this was true. We have shown that there is indeed an effect due to drug availability and cell density. Data in Figure 1 show that at ADM doses generally used (0–5 μg ADM $ml^{-1}$) in experiments when treating cells *in vitro*, the cell number can be an important factor in determining the clonogenic fraction; $2.5\times10^5$ cells $ml^{-1}$ gave a surviving fraction of 0.025 whereas $10^6$ cells $ml^{-1}$ gave a surviving fraction of 0.2, a difference of one decade, when treated with medium containing 2 μg $ml^{-1}$ ADM. When attempting to compare the sensitivities of clonogenic cells in monolayers of exponentially growing and plateau phase cultures, where the cell number present in the latter culture is at least one hundred times that of the former we now believe that it is important to take into account the effect of cell number in relationship to drug dose. Durand & Olive (1981) have also reported that ADM uptake is highly dependent on cell density and method of cell growth. Consequently we conclude that it is more representative to express the exposure drug dose as $\mu$g per $10^6$ cells.

Having established how best to represent our results and that FCM was giving a fair comparison of ADM levels in exponentially growing and plateau phase cells, we were able to draw some conclusions as to the response of the cells in these two proliferative states to ADM.

A similar range of exposure doses were given to both exponentially growing and plateau phase cells. Figure 3 indicates plateau phase cells were more sensitive over the dose range 0–10 $\mu$g per $10^6$ cells. Figure 5 shows that over that dose range a greater quantity of ADM was taken up by plateau phase cells. This indicates plateau phase cells have a far greater ability to accumulate ADM than exponentially growing cells but are less sensitive to a change in internal drug concentration. This point is demonstrated in Figure 4 where, for a given amount of internal ADM, plateau phase cells are far less sensitive than exponentially growing cells and the slope of their survival curve considerably less than that for exponentially growing cells.

Our results agree with the findings of Bhuyan *et al.* (1981) in that intracellular drug concentration may not account for the sensitivity of the cells to a

**Figure 5** FCM measurement of intracellular ADM fluorescence (absorbed dose) plotted against exposure dose of ADM. Open symbols, exponentially growing cells (4 replicate experiments); closed symbols, plateau phase cell (5 replicate experiments).

particular agent. Bhuyan *et al* (1981) found that in CHO cells treated with the anthracycline, 7-*con-O*-Methylnogarol, exponentially growing cells were more sensitive than plateau phase cells with the same internal drug concentration. Sutherland *et al* (1979) published data from exponentially growing and plateau phase EMT6 cells in monolayer treated with ADM and present the results as surviving fraction against internal drug concentration. They report only a small difference in sensitivity between the two proliferative states but they did not treat the plateau cells with the same range of dose of ADM as reported in this paper. Our results are similar to those of Sutherland *et al* (1979) over the range of dose of ADM that was used.

The differences in ADM uptake found in exponentially growing cells and plateau phase cells are likely to be primarily due to differences in the membrane permeability/drug export in the two proliferative states. Cell membrane differences have been shown to alter the uptake of ADM (Li & Hahn, 1978) and these data would suggest that plateau phase cells have more permeable membranes than exponentially growing cells.

The observation that, for a given amount of intracellular ADM, exponentially growing cells were far more sensitive to ADM is less easy to explain. We have demonstrated that the toxicity of ADM is not due simply to the total amount of ADM associated with the cell, therefore the ADM must be acting on a sensitive target that is preferentially expressed by exponentially growing cells, but not plateau phase cells. ADM is thought to act on several different sites within the cell (Bachur, 1975) and we have preliminary data suggesting that up to 45% of the ADM fluorescence for exponentially growing cells is associated with RNA (Smith and Chambers, unpublished data). Since exponentially growing cells have more RNA than plateau phase cells (Watson & Chambers, 1977), it is possible that the RNA content may play a role in ADM cytotoxicity.

Tritton (1982) has shown that the cell surface may be an important site for the cytotoxic action of ADM. If this is so the ratio of cell number to drug concentration will still be a factor in determining cell survival. However he does not rule out that the administration of free ADM affects cell viability through DNA intercalation. We have shown that in this case it is important to measure intracellular ADM concentrations in order to draw conclusions as to the relative sensitivities of cells to the drug.

### References

BACHUR, N.R. (1975). Adriamycin (NSC-123127) pharmacology. *Cancer Chemother. Rep.*, **6**, 153.

BARRANCO, S.C. & NOVAK, J.K. (1974). Survival responses of dividing and nondividing mammalian cells after treatment with hydroxyurea, arabinosylcytosine, or adriamycin. *Cancer Res.*, **34**, 1616.

BHUYAN, B.K., McGOVERN, P.J. & CRAMPTON, S.L. (1981). Intracellular uptake of 7-*con-O*-methylnogarol and adriamycin by cells in culture and its relationship to cell survival. *Cancer Res.*, **41**, 882.

DURAND, R.E. (1981). Flow cytometry studies of intracellular adriamycin in multicell spheroids *in vitro*. *Cancer Res.*, **41**, 3495.

DURAND, R.E. & OLIVE P.L. (1981). Flow cytometry studies of intracellular adriamycin in single cells *in vitro*. *Cancer Res.*, **41**, 3489.

GANAPATHI, R., REITER W. & KRISHAN, A. (1982). Intracellular adriamycin levels and cytotoxicity in adriamycin-sensitive and adriamycin-resistant P388 mouse leukemia cells. *J. Natl Cancer Inst.*, **68**, 1027.

KRISHAN, A. & GANAPATHI, R. (1980). Laser flow cytometric studies on the intracellular fluorescence of anthracyclines. *Cancer Res.*, **40**, 3895.

LI G.C. & HAHN G.M. (1978). Ethanol-induced tolerance to heat and adriamycin. *Nature*, **274**, 699.

SCHWARTZ, H.S. (1973). Fluorometric assay for daunomycin and adriamycin in animal tissues. *Biomed. Med.*, **7**, 396.

SUTHERLAND, R.M., EDDY, H.A., BAREHAM, B., REICH, K. & VANANTWERP, D. (1979). Resistance to adriamycin in multicellular spheroids. *Int. J. Radiat. Oncol. Biol. Phys.*, **5**, 1225.

TRITTON. T.R. & YEE, G. (1982). The anticancer agent adriamycin can be actively cytotoxic without entering cells. *Science*, **217**, 248.

TWENTYMAN, P.R. & BLEEHEN, N.M. (1975). Changes in the sensitivity to cytotoxic agents occurring during the life history of monolayer cultures of a mouse tumour cell line. *Br. J. Cancer*, **31**, 417.

TWENTYMAN, P.R., WATSON, J.V., BLEEHEN, N.M. & ROWLES, P.M. (1975). Changes in cell proliferation kinetics occurring during the life history of monolayer cultures of a mouse tumour cell line. *Cell Tissue Kinet.*, **8**, 41.

WATSON, J.V. (1980). Enzyme kinetic studies in cell populations using fluorogenic substrates and flow cytometric techniques. *Cytometry*, **1**, 143.

WATSON, J.V. & CHAMBERS, S.H. (1977). Fluorescence discrimination between diploid cells on their RNA content: A possible distinction between clonogenic and non-clonogenic cells. *Br. J. Cancer*, **36**, 592.